# Fracture Patterns in Craniofacial Gunshot Wounds: A Seven-Year Experience

**DOI:** 10.3390/cmtr18020023

**Published:** 2025-04-01

**Authors:** Gabriela G. Cruz, Sameer H. Siddiqui, David Z. Allen, Kunal R. Shetty, Sean P. McKee, Brady J. Anderson, Mark Knackstedt, W. Katherine Kao, Tang Ho

**Affiliations:** 1 John P. and Katherine G. McGovern Medical School, The University of Texas Health Science Center in Houston, Houston, TX 77030, USA; brady-anderson@uiowa.edu (B.J.A.); mark.knackstedt@lsuhs.edu (M.K.); 2 Department of Otorhinolaryngology–Head and Neck Surgery, McGovern Medical School at the University of Texas Health Science Center, Houston, TX 77030, USA; sameer.h.siddiqui@uth.tmc.edu (S.H.S.); david.allen@uth.tmc.edu (D.Z.A.); kunal.r.shetty@uth.tmc.edu (K.R.S.); wee.tin.k.kao@uth.tmc.edu (W.K.K.); tang.ho@uth.tmc.edu (T.H.); 3 Massachusetts Eye and Ear Infirmary, Department of Otolaryngology, Boston, MA 02114, USA; smckee1@meei.harvard.edu; 4 Department of Otolaryngology, University of Iowa, Iowa City, IA 52242, USA; 5 Department of Otolaryngology/HNS, Louisiana State University Health-Shreveport, Shreveport, LA 71103, USA

**Keywords:** gunshot wounds, facial trauma, facial fractures, level 1 trauma, surgical repair

## Abstract

Objective: To characterize facial fracture patterns and understand predictors of surgical repair and LOS with the objective of assisting providers in managing and understanding these complex injuries. Study Design: This is a retrospective cohort chart review study. Methods: A retrospective review was conducted for patients admitted with gunshot wounds (GSWs) to the head, neck, or face between January 2013 and March 2020 at a level one trauma tertiary care hospital. Univariate and multivariate analysis were performed to identify associations with surgical repair and LOS. Results: Of the 578 patients with head, neck, or facial GSWs, 204 survived and sustained facial fractures. The maxilla (*n* = 127, 62%), orbit (*n* = 114, 55%), and mandible (*n* = 104, 51%) were the most fractured. Operative rates differed by location (*p* < 0.001) with highest rates for fractures involving the mandible (76%). In univariate analysis, overall facial fracture surgery was associated with transfacial injuries; mandible, palate and nasal fractures; tracheostomy; gastrostomy tube placement; ICU admission; and a longer-than-24 h ICU stay (all *p* < 0.05). In multivariate analysis, predictors of surgical repair included a length of stay greater than 3 days (OR 2.9), transfascial injury (OR 3.7) and tracheostomy placement (OR 5.1; all *p*-values < 0.05), while nasal and mandible fractures were also associated with overall operative repair (OR 2.5 and 9.3, respectively; *p*-value < 0.05 for both). Univariate analysis showed that among patients with GSW injuries who underwent facial plastic reconstructive surgery (FPRS) with comorbid serious polytrauma, the inpatient LOS was predicted solely by the presence of subarachnoid, subdural and intracranial hemorrhage (*p*-value < 0.005). Subsequent multivariate analysis found that the only predictor for greater hospital LOS for patients who underwent surgical repair was earlier timing to FPRS of less than five days (OR 0.17) and placement of a gastrostomy tube (OR 7.85). Conclusions: Managing facial fractures in GSW patients requires complex medical decision making with a consideration of functional and esthetic outcomes in the context of concomitant injuries and overall prognosis. Certain characteristics such as ICU admission, longer hospital stay, trajectory of GSW, tracheostomy placement, and specific operative locations are associated with higher rates of operative repair. Inpatient hospitalization LOS for patients who underwent FPRS was predicted by timing from admission to surgical repair.

## 1. Introduction

Firearm injuries have been described as a public health issue that disproportionately affects racial and ethnic minorities and socioeconomically disadvantaged groups [1]. Gunshot wounds (GSWs) to the face portend high rates of morbidity and mortality, requiring prompt multidisciplinary intervention to improve functional, cosmetic, and psychological outcomes [2]. Facial fractures have been reported in up to 67% of GSWs to the face, but management often falls secondary to concomitant life-threatening injuries to the airway, head and neck vasculature, or central nervous system [2,3,4]. After initial stabilization and management of comorbid injuries, craniofacial trauma must be addressed to ensure maxillomandibular opening (MMO), occlusion, and facial symmetry [5,6].

Compared to other etiologies of facial fractures, GSWs have been associated with higher severity scores and likelihood of panfacial fracture patterns [7]. While many patients die following craniofacial GSWs, typically due to devastating neurologic injuries, those who survive often require extensive medical, surgical, and psychological treatments [1,5,8] The primary goal of the craniofacial surgeon is to optimize functional and esthetic outcomes. Initial management generally involves irrigation as well as debridement of devitalized tissues, followed by surgical fixation by means of open or closed reduction with internal or external fixation, and ranges from maxillomandibular fixation (MMF) to free tissue transfer for reconstruction, the optimal surgical timing of which is still contested [5,6,9,10].

Despite the well-established relationship between GSWs and facial fractures, there is a paucity of literature describing fracture frequency and management strategies specifically following GSWs. Previous retrospective studies are limited by small sample sizes attributed to the “uncommon” occurrence of facial GSWs [3,4,5,7], whereas large database studies lack detailed patient-level information [1,11]. The objective of this paper was to examine trends in facial fractures and management patterns following craniofacial GSWs at a high-volume trauma center in the United States and identify factors predictive of operative repair and hospital LOS.

## 2. Methods

This retrospective chart review was conducted with the approval of the Institutional Review Board of The University of Texas Health Science Center in Houston (HSC-GEN-13-0325). The Memorial Hermann Texas Level 1 Trauma Registry was used to identify patients that presented with an injury involving the head, neck, or face secondary to a firearm in combination with a severity Abbreviated Injury Scale (AIS) score greater than 0 between 1 January 2013 and 31 March 2020. Patients 18 years of age or older with a facial fracture secondary to a GSW involving the head, neck, or face met the inclusion criteria for this study. Patients without a facial fracture or those who had deceased prior to definitive repair of the facial fracture were excluded.

### 2.1. Data Abstraction

Emergency, inpatient, and operative records were reviewed to characterize facial fracture patterns and surgical management. Data abstracted included demographic information, facial fracture location, concomitant injuries, mechanism of injury, hospital course, surgical management, longitudinal follow-up, and death. Facial fractures were recorded as binary responses for each anatomic subsite; a comminuted fracture of the mandible would thus be counted as a single fracture. Transfacial injuries were defined as violating facial soft tissue and skeletal structures without affecting the cranial vault or intracranium.

The primary outcome was operative repair of the facial fracture. A binary classification system was used to identify fractures that underwent surgical repair by open or closed reduction, with or without fixation. For mandible fractures, surgical repair was defined as open reduction with internal fixation (ORIF), open reduction with external fixation (OREF), maxillomandibular fixation (MMF), or a combination of these methods. Secondary outcomes included demographic factors, fracture subsite, concomitant injuries, hospital course, and management factors associated with surgical repair.

### 2.2. Statistical Analysis

Comparisons were made between categorical variables using chi-squared tests and univariate logistic regression [12]. We utilized a student *t*-test for continuous variables after confirming normality of the data using Shapiro–Wilk tests. We determined a *p*-value of 0.05. Variables with a *p*-value below 0.20 on univariate regression analysis were included in a stepwise, forward multiple logistic regression analysis to develop a generalized linear model of factors predicting surgical repair. The data were split, trained, and tested using this model, and a receiver operating characteristic curve was calculated to demonstrate the fit [13]. Figures and statistical analysis were produced utilizing R (Vienna, Austria) [12,13,14].

## 3. Results

### 3.1. Demographics

**Figure 1:**
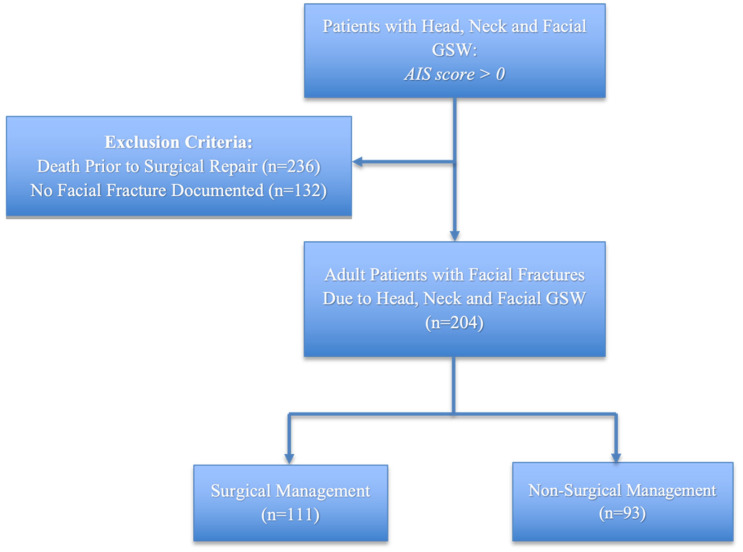
Study design and inclusion criteria.

Records were reviewed for 578 patients presenting with GSWs involving the head, neck, or face during the study period. Among these, 236 deceased prior to evaluation for surgical repair and 132 did not sustain a facial fracture, leaving a total of 204 surviving patients with facial fractures. Of these, 168 (82.8%) were male (Table 1). Racial and ethnic group representations included 35% (*n* = 71) Non-Hispanic White, 37% (*n* = 75) Non-Hispanic Black, 21% (*n* = 42) Hispanic, 4% (*n* = 8) Asian, and 4% (*n* = 8) unknown. Most patients were between 18 and 50 (*n* = 177, 87%). The average age of this cohort was 34 years old (standard deviation was 13.6). One third of our study were privately insured (*n* = 67, 32%), and another third were insured by publicly funded sources (*n* = 67, 33%) or lacked insurance (*n* = 60, 29%) altogether. There were 111 patients who underwent surgical management of facial fractures for an overall operative rate of 54.4% (Figure 1). Surgical repair was not associated with age, gender, race/ethnicity, or insurance type (all *p* > 0.05). In this cohort, solitary GSWs (*n* = 93, 46%) were the most common mechanism followed by self-inflicted GSWs (*n* = 60, 30%) and multiple GSWs (*n* = 50, 25%). No association was noted between the number of GSWs and operative repair (*p* > 0.05). The median number of fracture sites was 3 (IQR 1-5).

### 3.2. Fracture Location

**Figure 2:**
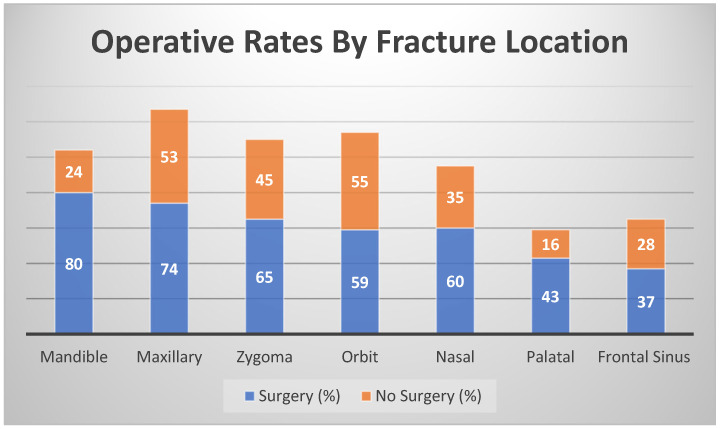
Operative rates by fracture location.

Facial fractures most impacted the maxilla (*n* = 127, 62%), followed by the orbit (*n* = 114, 56%), zygoma (*n* = 110, 54%), mandible (*n* = 104, 51%) and nasal regions (*n* = 95, 46%) (Figure 2). Fractures of the frontal sinus (*n* = 65, 32%) and palate (*n* = 59, 29%) were the least common. The rate of surgical repair was associated with fracture location (*p* < 0.001), with increased operative rates for fractures involving the mandible (80/104, 76%), palate (*n* = 43, 72%), nasal region (*n* = 60, 63%), zygoma (65/110, 59%), and maxilla (74/127, 58%). The lowest surgical repair rates involved the frontal sinus (37/65, 56%) and orbit (59/114, 51%; Figure 2). Among the mandible fractures, the technique for repair most often involved MMF (*n* = 30, 37%), ORIF (*n* = 22, 27%), a combination of ORIF and MMF (*n* = 9, 11%), a combination of OREF (*n* = 5, 6%), or a combination of OREF and ORIF (*n* = 13, 16%). Operative repair was more likely to be performed in patients with fractures of both the zygoma and maxilla (63/97, 65%, *p* < 0.05) and concomitant nasal and zygoma fractures (48/69, 70%, *p* < 0.05).

### 3.3. Univariate and Multivariate Analysis

Univariate analysis demonstrated that facial fractures associated with transfacial injuries (*n* = 135, 66% of cohort) had higher rates of surgical repair (OR 6.1, *p*-value < 0.05). Further, admissions to an intensive care unit (ICU; *n* = 141, 69%) and stays that were longer than 24 h (4%, *n* = 9) were both associated with higher rates of surgical repair (OR 2.3 and 17.6, *p*-values < 0.05 for both). Operative repair rates were also significantly higher among patients who underwent a tracheostomy (35.2%, *n* = 72) or gastrostomy tube placement (30%, *n* = 63; ORs 4.8 and 2.3, both *p*-values < 0.05) (Table 2).

**Figure 3:**
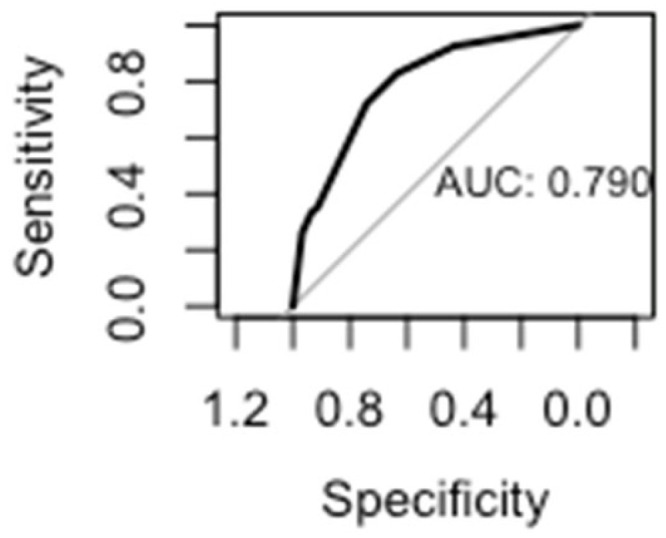
Regression model area under the curve.

A multivariate stepwise logistic regression model revealed increased odds of surgical repair for fractures involving the mandible (OR 9.3, 95% CI 4.7–19.3) and nasal bones (OR 2.5, 95% CI 1.13–5.9). Other factors that increased the odds of surgical repair included a length of stay greater than three days (OR 2.9, 95% CI 2.5–31.2), transfacial trajectory (OR 3.7, 95% CI 1.76–8.0), and tracheostomy placement (OR 5.1, 95% CI 1.7–19.5) (Table 3). After a test–train split of the data, the regression model incorporating the significant factors yielded an area under the curve of 0.79 (Figure 3).

### 3.4. Univariate and Multivariate Analysis of Length of Stay in Patients Who Underwent Facial Fracture Repair

Univariate analysis demonstrated that among comorbid injuries which included subarachnoid, subdural and intracranial hemorrhage (SAH/SDH/ICH), cervical spine injury (CSI), great vessel injury (GVI) and pharyngeal/laryngeal injury, only SAH/SDH/ICH was significantly associated with a greater LOS among patients who underwent FPRS (*p*-value < 0.005). Among all patients who underwent the FPRS procedure within five days of hospital admission, the average LOS was significantly shorter than the LOS of patients who underwent FPRS more than five days after hospital admission (15.2 versus 29.1 days *p*-value < 0.00005).

Multivariate analysis controlling for all factors elicited that early FPRS repair (within 5 days of admission) was shown to have a positive impact on LOS (OR 0.17, 95% CI 0.04–0.85, *p*-value < 0.05). Increased odds for an LOS greater than thirty days was also found when days to FPRS exceeded five days (OR 0.17, 95% CI 0.03–0.85, *p*-value < 0.05). The only other concomitant factor that increased the odds of a longer LOS (greater than 21 days) among patients who underwent FPRS was gastrostomy tube placement (OR 7.85, 95% 1.7–46.41, *p*-value < 0.05). None of the other comorbid injuries analyzed (SAH/SDH/ICH, CSI, GVI, pharyngeal and laryngeal injury) were found to have an association with LOS.

## 4. Discussion

The absence of an established, high-volume literature base on clinical assessment and management of facial GSWs left a gap in understanding of how various factors, including fracture sites, concomitant injuries, and hospital course, impact care. Considering the high morbidity and mortality rate of GSWs to the face, 67% of which involve facial fractures, our study sought to understand the clinical practice patterns of facial fracture injuries in GSW victims and evaluate their inpatient course [2,3,4]. We wanted to specifically assess which polytraumatic injuries and associated factors ultimately lead to inpatient surgical repair and extended hospitalizations.

To date, this is one of the largest papers to examine factors associated with the surgical repair of facial fractures specifically following facial gunshot wounds [4,15]. This study identified novel predictive factors including transfacial trajectories, palatal fractures, concomitant tracheostomy procedures, ICU stay, and a longer-than-48 h admission following a craniofacial GSW, as well as factors associated with conservative management (e.g., cervical spine injury). Our study included all adult patients that presented to one of the busiest level 1 trauma centers in the United States in one of the country’s most populated cities between 1 January 2013 and 31 March 2020.

In accordance with these devastating injuries, we found an astonishing high mortality rate of over 40% (*n* = 236) of our initial cohort. Similar to previous studies, our study population was predominantly male (82%) and between the ages of 18 and 50 years [1,4,11]. In our study, the rate of operative repair (54.4%) was within the published range of 39% to 64 [15,16]. The mandible is among the most commonly fractured bones following a facial GSW and requires high rates of surgical intervention, likely due to its critical role in mastication [2,4,15,17]. Mandibular involvement in facial fractures has been associated with increased odds of repair in both adult and pediatric populations; however, these studies examined fractures secondary to all causes without highlighting the particularly devastating injuries associated with GSWs [18,19]. Ballistic studies have shown that the brittle cortical bone of the mandible is more likely to shatter following projectile trauma, causing severe comminution that requires extensive reconstruction [20,21].

Perhaps not surprisingly, mandibular fractures (OR 7.3) were independent predictors of repair in our study, and the extensive destructive nature of these injuries was highlighted by the exceedingly high rate of external fixation performed in nearly one-quarter of cases. External fixation is a known treatment for highly comminuted fractures [22,23]. In one 10-year series of 196 comminuted mandible fractures, external fixation was employed in only 17 cases (8.6%), 11 of which were GSWs, whereas the rate of external fixation in our study exceeded 20% [24]. The optimal surgical approach to mandibular damage in the setting of GSWs is controversial but generally involves debridement, accompanied or followed by repair via open or closed reduction, with or without interdental fixation, with the goal of restoring occlusion and adequate jaw mobility [10]. For large soft- or bony-tissue deficits, free nonvascularized bone grafts or microvascular tissue transfers may be required to close wounds and permit future deglutition, as observed in approximately 10% of our cohort [24,25]. In a large study such as ours with many surgically treated mandible fractures, it would be exceedingly useful to evaluate long-term differences in malunion and occlusion for patients treated with internal rigid fixation compared to intermaxillary fixation.

In our study, the maxilla was the most fractured site (62%) but did not have a relative higher operative rate (58%), particularly in comparison to other fracture locations. The maxilla assists in achieving occlusion, supporting the upper airway, and providing contour to the face. Previous studies have shown a high rate of maxillary fractures and repair following GSWs, which is in contrast with the results found in our cohort [2]. Although typical fracture patterns seen in blunt force trauma, such as naso-orbital-ethmoid (NOE), zygomaticomaxillary (ZMC), and LeFort, are not adhered to in penetrating injuries and were not specifically categorized in our study, we also found that patients with both zygomatic and maxillary fractures (*n* = 97), 65% (*n* = 63) had some form of facial fracture repair which was higher relative to isolated maxillary or zygomatic fractures. This finding may reflect the need for the restoration of lateral buttress support, akin to ZMC fractures [26]. Higher operative rates were also noted for combined nasal and orbital fractures, akin to NOE fractures, although statistical significance was not reached.

In our cohort, transfacial injuries were independently associated with surgical repair (OR 6.2). Fractures limited to the facial skeleton without intracranial involvement are rarely fatal, whereas intracranial involvement portend a high predilection for death prior [1,2]. Even when non-fatal, concomitant injuries may take priority over facial fracture repair or render it unsafe [2,3,4], as suggested in our cohort by the lower rates of fracture repair in patients with cervical spine injury, skull or temporal bone fractures, and lower—although not statistically significant—rates in patients with intracranial injury or neurosurgical intervention.

In this cohort, a LOS longer than 72 h was associated with an increased likelihood of facial fracture repair. As most of the mortality due to facial GSWs has been reported to occur within one day [8], our findings may reflect the importance of both survival and hospitalization for greater than 72 h after fracture repair. It also highlights the complexity of craniofacial GSWs, which are often associated with lengthy hospital stays [1,8] in contrast to fractures by other mechanisms that may be managed on an outpatient basis [27]. The association with LOS may also indicate a lengthened timetable for repair in those admitted for longer periods, and the opportunity for repairs to be coordinated with other operations under a single general anesthetic.

The higher rates of fracture repair among patients receiving gastrostomy placement (*p* = 0.009) and tracheostomy (*p* < 0.001), an independent predictor in addition to length of stay, may reflect improved survival odds, as high rates of tracheostomy and gastrostomy have been associated with increased survival following self-inflicted GSWs [8,28,29,30]. Traumatic facial fractures may cause airway compromise as a result of anatomic disruption or obstruction by blood or debris, and in the acute setting, airway establishment with either an endotracheal tube or surgical airway takes precedence over craniofacial injuries and remains the highest priority [2,16,29,31,32]. The observed rate of tracheostomy in our cohort (27%) was consistent with the literature, as fractures due to GSWs have been associated with particularly high rates of surgical airway management in up to 50% of patients [25,33].

The rate of CSI in our cohort was 6%, consistent with reported values of 5–10% in patients with facial GSWs [16,34]. CSI can cause life and limb-threatening complications, including quadriplegia, with treatment ranging from cervical collar immobilization when asymptomatic to surgical decompression and rigid fixation for injuries with neurologic deficits [35]. While cervical precautions from the spine team can be safely adhered to intraoperatively and do not necessarily preclude the repair of facial fractures, patients with CSI are often in a critical condition with a poor clinical prognosis, and conservative management may be preferred to avoid catastrophic spinal complications. However, delays in definitive treatment may lead to secondary healing of wounds, thus complicating the eventual facial reconstruction [34]. Notably, in our cohort, CSI was not independently associated with significantly decreased operative repair (OR 0.39). Nonetheless, although not addressed herein, future studies of this subgroup that examine craniofacial complications such as infection, malunion, non-union, and delayed healing in this population, in addition to functional outcomes, would be informative.

Length of hospital stay, when extended, can come with many medical and financial complications to both the patient and hospital. Prior research has indicated that prolonged inpatient stays can increase a patient’s risk for hospital-acquired infections and falls, which would be especially debilitating for patients that are recovering from major FPRS [36]. Additionally, patients with extended hospital stays cause greater medical costs for themselves and the hospital, creating pressure on patients recovering from FPRS. However, in patients who are healthy and stable enough to undergo surgery, we were able to determine that those who underwent earlier facial fracture repair (within 5 days of admission) had a less overall LOS. Moreover, timing to surgical repair, even when controlled for all other factors, was the only factor other than gastrostomy tube placement that was associated with an earlier discharge in multivariate analysis, regardless of comorbid injuries including intracranial trauma or individual and specific fracture sites. Given the added benefits of minimizing an unnecessary extension of hospital stay, including decreased hospital acquired medical complications and medical cost and expenditure, these findings could guide trauma surgeons in their clinical assessment of patients with GSW facial fractures. Conducting FPRS repair more acutely for these patients could prove worthwhile for the overall impact on the patient’s recovery and the goal of minimizing unnecessary resource allocations for hospital systems.

This study is limited by its retrospective utilization of medical records which are prone to errors in documentation, coding, or review. To mitigate, these records were examined by physicians experienced in the management of craniofacial trauma. While the large sample size may increase external validity, this cohort reflects a single institution’s experience and may not generalize to other populations. Although various craniofacial fracture patterns were described, our study did not further delineate anatomic subsites or stratify by degree of destruction. Furthermore, we did not investigate the various repair techniques or examine perioperative complications or outcomes. Although known to affect wound characteristics, information pertaining to the classification, caliber, proximity, and velocity of the firearm injuries could not be ascertained from the medical record [10,20]. Lastly, the results of our study only reflect treatment received at our institution, which may underestimate the true rate of repairs if any were subsequently performed elsewhere.

Future studies could be manyfold. Investigations into the systemic drivers of racial, ethnic, and socioeconomic disparities in gunshot violence is necessary. Future inquiry into the perioperative outcomes and complications of craniofacial trauma could also provide a valuable insight into additional considerations for the reconstructive surgeon. A deeper investigation into the natural course of trauma as a disease in the context of craniofacial trauma and its related medical illnesses would also be of benefit as these patients often endure chronic physical, emotional, psychological, and financial burdens.

## 5. Conclusions

Managing facial fractures in GSW patients requires complex medical decision making with a consideration of functional and esthetic outcomes in the context of concomitant injuries, LOS, and overall prognosis. Patients who had mandible or nasal fractures more often had inpatient repair compared to other facial subsites. Further, patients who had a tracheostomy placement and those who had a prolonged critical care stay more often underwent repair.

## Figures and Tables

**Table 1:** Demographic factors and facial fractures.

Variable	*N*	%	Operative Repair %	OR 95% CI
Male	168	82.8	53	
Female	35	17.1	57	1.14 (0.52–2.6)
LOS > 1 day	186	91.1		11.2 (2.5–103.4)
Non-Hispanic White	71	34.8	53.5	
Non-Hispanic Black	75	36.7	52	
Hispanic	42	20.5	55	
Asian	8	3.9	75	
Unknown	8	3.9	62	
Private Insurance	67	32.8	52	
Medicare Insurance	9	4.41	77	
Medicaid Out	1	0.49	0	
Medicaid IN	21	10.2	43	
Self-Pay	60	29.4	56	
Crime Victims	38	18.6	63	
Other	8	3.9	25	

χ^2^ results for all demographics were not significant, *p* > 0.05.

**Table 2:** Injury hospital factors and operative repair.

Variable	Total	No Surgery	Surgery	*p*-Value	OR	5% CI OR	95% CI OR
* Mandible Fractures	104	24	80	4.52 × 10^−11^	7.3	3.8	14.5
* Palatal Fractures	59	16	43	0.001	3.02	1.5	6.3
* Transfacial Injuries	135	46	89	6.1 × 10^−8^	6.2	2.96	13.5
* Nasal Fractures	95	35	60	0.024	1.94	1.07	3.56
Transcranial Injuries	12	11	1	0.3	0.53	0.16	1.6
Maxillary Fractures	127	53	74	0.1919	1.5	0.82	2.78
Zygoma Fractures	110	45	65	0.16	1.5	0.83	2.72
Orbit Fractures	114	55	59	0.4	0.78	0.43	1.42
Temporal Bone Fractures	93	25	21	0.18	1.94	1.07	3.56
Frontal Sinus Fractures	65	28	37	0.65	1.159	0.62	2.2
Skull Fractures	69	34	35	0.46	0.8	0.43	1.49
Intracranial Fractures	44	22	22	0.61	0.8	0.39	1.65
Globe Injuries	56	32	24	0.058	0.52	0.27	1.03
* ICU Stay	141	55	86	0.009	2.3	1.2	4.47
ICU Stay > 24 h	9	1	8	0.009	17.6	1.4	1052
* Tracheostomy	72	16	56	8.86 × 10^−7^	4.8	2.4	9.955
* Gastrostomy	63	20	43	0.009	2.3	1.2	4.65
SAH/SDH/IDH	58	32	26	0.12	0.59	0.3	1.14
Cervical Spinal Injuries	18	12	6	0.082	0.39	0.11	1.2
Great Vessel Injuries	27	15	12	0.3	0.63	0.25	1.54
Pharynx/Larynx/Subglottic Injuries	22	6	16	0.07	2.4	0.86	7.9
Ophthalmology Consult	104	52	52	0.26	0.71	0.39	1.27
NSGY Consult	118	55	63	0.89	0.93	0.51	1.68
Cerebroangiogram	37	21	16	0.149	0.58	0.26	1.272
Psych Consult	69	25	44	0.052	1.8	0.97	3.5

* denotes statistical significance.

**Table 3:** Multivariate analysis of fracture location and hospital factors.

Multivariate Factor	Estimate	*z*	*p*	OR	2.5% CI OR	97.5% CI OR
* LOS > 3 days	1.0578	2.001	0.04539	2.8800259	1.0471754	8.4849732
* Transfacial Trajectory	1.39496	3.681	0.000232	3.6955242	1.7566235	8.0337512
* Tracheostomy	1.67517	2.732	0.006288	5.1000371	1.6557131	19.546377
ICU Stay	0.05116	0.126	0.899938	0.9693761	0.407843	2.229332
PEG	−0.77772	−1.271	0.20378	0.432366	0.1138146	1.3423036
* Nasal	0.9353	2.227	0.026	2.5	1.13	5.9
* Mandible	2.2252	6.207	5.39 × 10^−10^	9.3	4.7	19.3
Palatal	0.5001	1.114	0.27	1.6	0.69	4

* denotes statistical significance.

## Data Availability

The data presented in this study are available on request. The data presented in this study are available on request from the corresponding author due to privacy reasons.

## Abbreviations

The following abbreviations are used in this manuscript:

LOS	Length of stay
GSW	Gunshot wound
FPRS	Facial plastic reconstructive surgery
MMO	Maxillomandibular opening
MMF	Maxillomandibular fixation
ORIF	Open reduction with internal fixation
OREF	Open reduction with external fixation
SAH	Subarachnoid hemorrhage
SDH	Subdural hemorrhage
ICH	Intracranial hemorrhage
CSI	Cervical spine injury
GVI	Great vessel injury
NOE	Naso-orbital-ethmoid
ZMC	Zygomaticomaxillary
